# Understanding gender differences in reasoning and specific paradigm using meta-analysis of neuroimaging

**DOI:** 10.3389/fnbeh.2024.1457663

**Published:** 2025-01-07

**Authors:** Lina Chen, Zeqing Zheng, Jin Liang, Yuerui Lin, Qingqing Miao

**Affiliations:** ^1^School of Psychology, Capital Normal University, Beijing, China; ^2^Department of Education, Hengshui University, Hengshui, China; ^3^State Key Laboratory of Cognitive Neuroscience and Learning, Beijing Normal University, Beijing, China; ^4^China Institute of Marine Technology and Economy, Beijing, China; ^5^National Key Laboratory of Human Factors Engineering, Beijing, China; ^6^College of Foreign Languages and Literature, Northwest Normal University, Lanzhou, China

**Keywords:** reasoning, gender differences, meta-analysis, ALE, WCST

## Abstract

Reasoning is a fundamental cognitive process that allows individuals to make inferences, decisions, and solve problems. Understanding the neural mechanisms of reasoning and the gender differences in these mechanisms is crucial for comprehending the neural foundations of reasoning and promoting gender equality in cognitive processing. This study conducted an Activation Likelihood Estimation (ALE) meta-analysis of 275 studies, revealing that reasoning involves multiple brain regions, including the parts of frontal, parietal, occipital, temporal lobes, limbic system, and subcortical areas. These findings indicate that reasoning is a complex cognitive process requiring the coordinated activity of multiple brain regions. Additionally, 25 studies focusing on the Wisconsin Card Sorting Test (WCST) paradigm confirmed the importance of these regions in reasoning processes. The gender-specific activation results indicate that males and females utilize different neural networks during reasoning and WCST tasks. While significant differences exist in specific regions, the overall activation patterns do not show marked gender differences. Notably, females exhibit greater activation in the limbic system compared to males, suggesting that emotional states may play a more prominent role for females when engaging in reasoning tasks.

## 1 Introduction

Reasoning is an advanced cognitive process that enables individuals to make inferences, decisions, and solve problems. It is a crucial aspect of human intelligence, underpinning various daily activities from social interactions to conducting complex scientific analyses. Understanding the neural mechanisms behind reasoning helps to elucidate how the brain supports these higher cognitive functions, aligning with the goals of cognitive neuroscience to connect brain function with neural substrates (Poldrack, 2010). Consequently, research utilizing neuroimaging tools has emphasized the role of reasoning in analyzing social cognitive processes (Lieberman, 2010) and exploring the neural processes of reasoning (Calzavarini and Cevolani, 2022).

Numerous neuroimaging studies have investigated the neural correlates of reasoning (Melrose et al., 2007) found that the prefrontal-striatal circuit is involved in abstract reasoning. Other studies have shown that deductive reasoning activates parietal-occipital regions (Prado et al., 2011), and paradoxical reasoning activates the prefrontal/orbital frontal cortex. The prefrontal cortex (PFC) is crucial for both conditional and syllogistic reasoning, playing a key role in integrating information and managing cognitive tasks (Wertheim and Ragni, 2020). These studies used different concepts and paradigms of reasoning, leading to various identified brain activation regions. Nevertheless, activation of DLPFC and parietal regions during reasoning is widely recognized (Christoff et al., 2001; Kroger et al., 2002; Prabhakaran et al., 1997). The rostrolateral PFC may also significantly contribute to reasoning (Modi et al., 2018). A meta-analysis of fMRI studies highlights the collaboration between the PFC and parietal regions in supporting relational reasoning by processing and integrating complex information (Prado et al., 2011). And, during problem-solving, connectivity between the PFC and regions like the parietal lobe is essential for efficient reasoning and decision-making (Bartley et al., 2018).

Additionally, cognitive neuroscience research increasingly focuses on exploring gender differences in brain structure and function. Studies have demonstrated differences in brain activity and cognitive performance between men and women, highlighting the importance of considering gender factors in cognitive neuroscience research (Grady, 2008). There have been studies on gender differences in different reasoning tasks. For example, Prado et al. (2011) found that while males and females perform similarly on deductive reasoning tasks (e.g., syllogistic reasoning, conditional inferences), they show different brain activation patterns. Males tend to activate the left parietal and occipital regions more, whereas females are more likely to activate prefrontal regions. Gur et al. (2000) discovered that males activate more of the right parietal region during spatial reasoning tasks (e.g., mental rotation, spatial visualization), while females tend to activate the left prefrontal cortex. Furthermore, research has shown that females exhibit more significant activation in the medial prefrontal cortex (mPFC) and superior temporal sulcus (STS) during tasks involving emotion and social cognition, whereas males show more pronounced activation in the prefrontal cortex during tasks involving rational analysis (Schlaffke et al., 2015). Therefore, understanding the neural mechanisms of reasoning and the neural differences between gender is crucial for comprehending the neural foundations of reasoning and promoting gender equality in reasoning abilities.

This study aims to utilize existing neuroimaging data to conduct a comprehensive meta-analysis of the neural imaging studies on reasoning and its typical experimental paradigm, the Wisconsin Card Sorting Test (WCST), to elucidate gender-specific brain activation patterns in reasoning functions. The WCST is a well-established neuropsychological test that evaluates executive functions such as cognitive flexibility, problem-solving, and the ability to shift strategies in response to changing environmental contingencies (Grant and Berg, 1993). These functions are critical components of reasoning, as they require individuals to adapt and apply logical principles to solve novel problems. Our choice of the WCST was motivated by its robust ability to engage and measure the cognitive processes underlying reasoning, making it a valuable tool for investigating the neural mechanisms involved.

Using the Activation Likelihood Estimation (ALE) method, we systematically identify consistent activation patterns associated with reasoning tasks in males and females. First, ALE allows for the comprehensive aggregation of data from multiple neuroimaging studies, identifying consistent brain activation patterns across different datasets, which enhances the generalizability of findings (Eickhoff et al., 2012), also statistically robust, employing probabilistic modeling and permutation testing to ensure the significance of observed activation patterns, reducing the risk of false positives (Laird et al., 2005a). Turkeltaub et al. (2002) accounts for variability across studies, such as differences in scanner resolution and participant populations, by modeling activation foci as probability distributions. This makes it ideal for comparing sex-specific brain activation. Moreover, Laird et al. (2009) proposed a meta-analytic activation consistency mapping (MACM) method that can identify common activation patterns in functionally related regions, and ALE facilitates subgroup analysis, enabling the detection of distinct neural activation patterns between males and females, which is essential for uncovering sex-specific differences in reasoning (Kohn et al., 2014). Overall, ALE’s ability te data from diverse studies provides a more generalized and reliable understanding of the neural mechanisms underlying reasoning and their potential gender differences(Costafreda, 2012; Fox et al., 2005). This approach provides a robust and generalized understanding of the sex differences in the neural foundations of reasoning.

## 2 Methods

To verify the neural basis of reasoning, specifically the neural basis of reasoning across genders, the BrainMap database was queried using Sleuth version 3.0.4 (Fox et al., 2005; Laird et al., 2009; Laird et al., 2005b). Sleuth^1^ is a free, publicly available search tool that allows users to search the BrainMap database according to two different categories (Hill et al., 2014). As an important note in terminology in the literature and BrainMap database search, we chose to use “gender” rather than “sex” throughout our study. We formulated the following search criteria:

(1) Find researches on reasoning or using the WCST paradigm (e.g., Experiments → Behavioral Domain → Cognition → Reasoning).
(2) Studies reporting only activation results (e.g., Experiments → Activation → Activations (or Deactivations) Only).
(3) Studies using normal, healthy subjects (e.g., Experiments → Context → Normal Mapping).
(4) Studies using three searches for gender: only males or only females or no restrict for gender (e.g., Subjects → Gender → Females Only).

The global activation and deactivation coordinates of the search results were obtained. The comprehensive results for reasoning were shown as Table 1.

**TABLE 1:** The activation coordinates of the reasoning results from Brainmap.

	Search	Studies	Locations	Experiments	Conditions	Subjects
Activation	All studies	275	9974	358	1051	7390
	Female-specific	21	712	158	60	586
	Male-specific	90	2732	428	303	2203
Deactivation	All studies	29	373	60	101	444
	Female-specific	3	9	4	9	54
	Male-specific	7	89	12	22	99

In addition to these general reasoning findings, our search also identified a subset of studies that specifically utilized the WCST paradigm. These WCST-related studies reported activation patterns associated with this reasoning-based cognitive task. For the WCST paradigm, the results of activation were:

(1) All studies: 25 papers, 908 locations, 113 experiments, 83 conditions, 765 subjects.
(2) Female-specific studies: 4 papers, 91 locations, 23 experiments, 8 conditions, 157 subjects.
(3) Male-specific studies: 5 papers, 298 locations, 17 experiments, 16 conditions, 57 subjects.

For the WCST studies, in addition to the activation findings, the search identified 4 papers that reported deactivations (decreases in neural activity) associated with this reasoning task. These 4 WCST studies reported a total of 47 deactivation locations, 11 experiments, and 20 conditions, involving 44 participants. However, the BrainMap database did not contain any gender-specific data for these deactivation findings - the results were not stratified by male or female participants. The obtained results were then downloaded locally. GingerALE^2^ is a commonly used tool for coordinate-based meta-analysis. The ALE method, first proposed by Laird et al. (2005a) and further verified and improved by Turkeltaub et al. (2002), has been enhanced by Eickhoff et al. (2009) and implemented in the statistical toolbox GingerALE. Using the extracted coordinate values, we performed meta-analyses on the peak coordinate points for the different results using GingerALE. In addition, in the exploration of the neural mechanisms related to reasoning, the results of the image meta-analysis of reasoning were used as a reference, and the largest 5 ROIs were set for MACM.

In ALE meta-analysis, the False Discovery Rate (FDR) correction is a commonly used method for multiple comparison corrections. The specific calculation of FDR depends on the number of permutation tests performed (Laird et al., 2005a). In this study, we conservatively corrected ALE statistical images using FDR (FDR corrected *p*-value < 0.05) and set a minimum clustering threshold of 200 mm^3^. This correction method helps control the risk of Type I errors, allowing for more reliable identification of consistent and stable activation patterns in our integrated analysis. Additionally, it ensures that the discovered activation regions have high credibility.

For visualization, we utilized the Workbench^3^ software to present the neural activation images. Workbench is a powerful tool for viewing and analyzing neuroimaging data, allowing us to effectively illustrate the gender-specific neural activation patterns associated with reasoning.

## 3 Results

### 3.1 Brain activation regions and connectivity for reasoning in behavioral and paradigm domains

**FIGURE 1:**
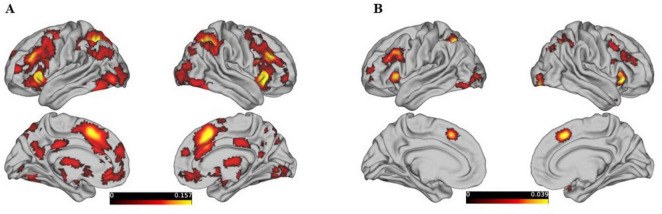
Shows the activation maps of different brain regions in both the left and right hemispheres, as well as medial and lateral views, without distinguishing by gender, threshold at *p* < 0.05, FDR-corrected. **(A)** Represents the activation map dominated by behavioral tasks, while **(B)** represents the activation map dominated by the WCST paradigm.

The ALE meta-analysis of 275 studies identified 10 clusters, with the largest cluster having a volume of 119,576 mm^3^ and containing 26 peak points, and the largest ALE value is 0.218. This comprehensive list of brain regions highlights the widespread involvement of frontal, parietal, occipital, temporal, limbic, and subcortical areas in supporting reasoning processes (Figure 1). The main regions include the left and right inferior frontal gyrus (BA9), middle frontal gyrus (BA9), and superior frontal gyrus (BA6); the inferior parietal lobule (BA40) and superior parietal lobule (BA7); the precuneus (BA7); the inferior occipital gyrus (BA18) and middle occipital gyrus (BA19); the fusiform gyrus (BA37); the anterior cingulate (BA24); and the parahippocampal gyrus. Detailed brain regions can be found in Supplementary Table 1. These areas collectively illustrate the extensive neural network engaged in reasoning.

The results of the 25 studies on the Wisconsin Card Sorting Test (WCST) are largely encompassed by the brain activation results related to reasoning, with the locations of peak points closely aligning with the peak locations of reasoning activation. There are 18 clusters in total, the largest of which has a volume of 11,944 mm^3^ and an ALE value of 0.051. Detailed brain regions can be found in Supplementary Table 2.

**FIGURE 2:**
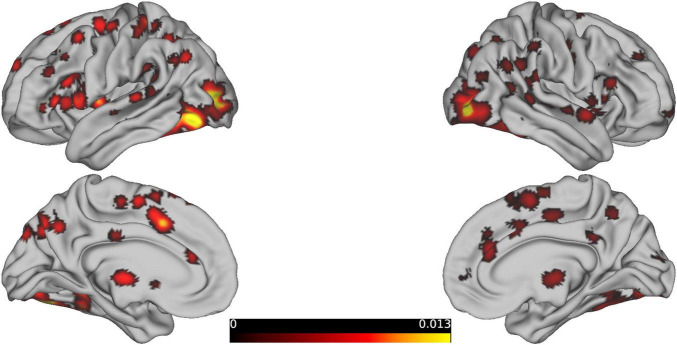
Presents the brain regions showing consistent activations associated with reasoning tasks.

By ALE meta-analysis of the experiments in BrainMap, the central MNI coordinates of the five largest clusters were used to create 5 mm ROI spheres and were entered into the BrainMap database to search for all activations reported within the boundaries of each ROI. Based on the results obtained, the MACM map of the five selected seed ROIs was calculated, and the activation focus closest to each seed ROI showed a significantly higher probability of co-activation (Figure 2).

**FIGURE 3:**
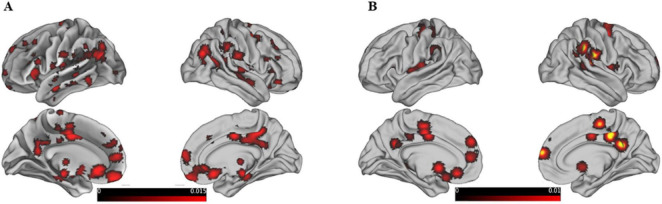
Shows the same content as the previous graph, except that this graph is the result of deactivation. **(A)** Shows the deactivation graph related mainly to reasoning-related behavioral tasks, and **(B)** shows the deactivation graph mainly based on studies using the WCST paradigm.

The meta-analysis of 29 studies identified 74 clusters, with the largest cluster having a volume of 20,464 mm^3^ and containing 14 peak activations with a maximum ALE value of 0.021(Figure 3A). These values are much lower than typical positive activations and differ from the patterns of positive activation, primarily involving BA 10, 32, 40, 39, and 31. The results from 4 studies using WCST, largely showed brain deactivations (Figure 3B). The peak coordinates of these deactivations were in close proximity to the peak locations of the reasoning-related deactivations. In total, there were 16 clusters, with the largest cluster having a volume of 19,712 mm^3^ and an ALE value of 0.011.

### 3.2 Gender-specific brain activation on behavioral and paradigm domains

**FIGURE 4:**
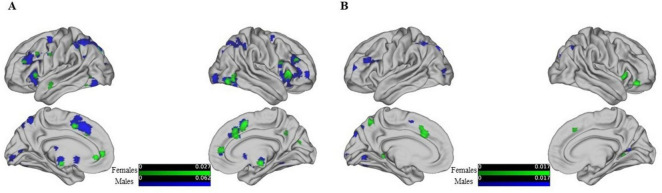
Shows the activation maps of different brain regions for males and females from left, right, medial, and lateral views, threshold at *p* < 0.05, FDR-corrected. Different colors are used to represent these differences, with varying shades indicating the degree of activation: green represents female brain activation maps, and blue represents male brain activation maps. **(A)** Shows behavior-driven activation maps, while **(B)** shows WSCT paradigm-driven activation maps.

Further analysis based on gender-specific activation patterns during reasoning and WCST tasks revealed distinct neural networks for males and females as shown in Figure 4A. The study highlights specific brain regions where different genders show significant neural activity related to reasoning. For females, notable activations include the right sub-lobar insula (BA 13), the left inferior parietal lobule (BA 40), and the right cingulate gyrus (BA 32). The largest cluster for females measures 1,664 mm^3^. For males, significant activations are observed in the left superior parietal lobule (BA 7), the left precuneus (BA 19), and the right inferior frontal gyrus (BA 46). The largest cluster for males measures 12,464 mm^3^.

The gender differences in WCST activation, showing in Figure 4B. In females, additional areas of activation include the left cingulate gyrus (BA 32), the right medial frontal gyrus (BA 47), and the bilateral parahippocampal gyrus (BA 19 and 30). In contrast, males show prominent activity in the left superior parietal lobule (BA 7) and the right precuneus (BAs 19 and 31). Detailed gender-specific reasoning and WCST activation maps can be found in Supplementary Tables 3–6. The findings emphasize the different neural bases involved in reasoning and WCST tasks for males and females, reflecting gender-specific neural networks engaged in these cognitive processes. Although less articles were found and analyzed, the small amount of data was not sufficient to support the authenticity of the results, so the results of deactivation were not reflected in gender differences.

### 3.3 Contrast of gender differences in activation

**FIGURE 5:**
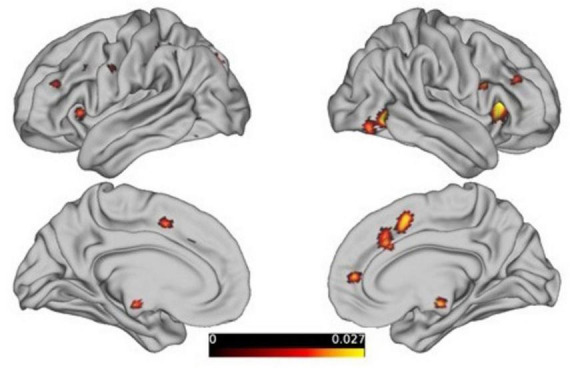
The contrast analysis of the resultant ALE maps from females to males, threshold at p < 0.05, FDR-corrected.

We also performed a quantitative comparison of the resulting ALE maps using the GingerALE program within the BrainMap environment to objectively determine the differences between male and female networks in a statistically sound manner. GingerALE achieves this by subtracting two ALE result images. Similar to traditional ALE analyses, GingerALE pools the coordinates from the original datasets and randomly assigns them into two new groups of the same size as the original datasets. These new pairings are then subtracted (i.e., using permutations to create and compare the null distribution of the real data). The resulting images are converted into z-score maps as shown in Figure 5.

The analysis of the “females minus males” results revealed that there are still 18 clusters with volumes exceeding 200 mm^3^ in the reasoning task (showed Table 2), primarily distributed in the prefrontal cortex and limbic system, as detailed in the figure below. However, the males minus females results and the gender differences driven by the WCST paradigm did not survive correction.

**TABLE 2:** Significant clusters (FDR *p* < 0.05) of contrast from females to males about reasoning revealed by the ALE analysis.

Cluster	Volume (mm^3^)	ALE	Weighted centre			Peaks	Peak MNI			Anatomical region
			***x***	***y***	***z***		***x***	***y***	***z***	
1	1664	0.035	34.5	24.4	3.5	1	34	24	6	Right Sub-lobar, Insula, Brodmann area 13
2	1536	0.033	−42.9	−43.1	45	1	−42	−42	46	Left Parietal Lobe, Inferior Parietal Lobule, Brodmann area 40
3	1240	0.029	5.3	25.7	34.8	3	8	24	32	Right Limbic Lobe, Cingulate Gyrus, Brodmann area 32
							4	26	38	Right Limbic Lobe, Cingulate Gyrus, Brodmann area 32
							−6	24	32	Left Limbic Lobe, Cingulate Gyrus, Brodmann area 32
4	1224	0.036	46.9	−62.5	−4.7	3	46	−58	−2	Right Temporal Lobe, Middle Temporal Gyrus, Brodmann area 37
							44	−70	−12	Right Occipital Lobe, Fusiform Gyrus, Brodmann area 19
							50	−66	−10	Right Temporal Lobe, Fusiform Gyrus, Brodmann area 37
5	712	0.028	48.2	10.6	24.6	1	48	10	26	Right Frontal Lobe, Inferior Frontal Gyrus, Brodmann area 9
6	704	0.026	8.8	13	48	1	10	12	50	Right Frontal Lobe, Medial Frontal Gyrus, Brodmann area 6
7	568	0.021	−48.5	−0.3	33.8	2	−52	0	32	Left Frontal Lobe, Precentral Gyrus, Brodmann area 6
							−44	−4	36	Left Frontal Lobe, Precentral Gyrus, Brodmann area 6
8	560	0.029	−19.8	6.6	4.2	1	−20	6	4	Left Sub-lobar, Lentiform Nucleus, Putamen
9	472	0.023	43.6	−46	45.8	2	42	−46	42	Right Parietal Lobe, Inferior Parietal Lobule, Brodmann area 40
							46	−46	50	Right Parietal Lobe, Inferior Parietal Lobule, Brodmann area 40
10	432	0.025	16.2	7.6	5.8	1	16	8	6	Right Sub-lobar, Caudate, Caudate Body
11	360	0.022	47.3	38.3	25.1	1	48	42	28	Right Frontal Lobe, Superior Frontal Gyrus, Brodmann area 9
12	328	0.025	−48.1	−59	−12.9	1	−48	−58	−14	Left Temporal Lobe, Fusiform Gyrus, Brodmann area 37
13	328	0.025	−22.1	−57.4	43.7	1	−22	−58	44	Left Parietal Lobe, Precuneus, Brodmann area 7
14	288	0.027	−45.8	38.4	22.2	1	−48	40	22	Left Frontal Lobe, Middle Frontal Gyrus, Brodmann area 46
15	272	0.022	−34.6	20.7	1.5	1	−34	20	2	Left Sub-lobar, Insula, Brodmann area 13
16	256	0.022	−23.5	−79.3	37.4	1	−24	−80	36	Left Occipital Lobe, Precuneus, Brodmann area 31
17	240	0.024	11.4	48.7	8	1	10	50	8	Right Frontal Lobe, Medial Frontal Gyrus, Brodmann area 9
18	240	0.023	−5.2	6.7	46.4	1	−6	6	46	Left Limbic Lobe, Cingulate Gyrus, Brodmann area 24

## 4 Discussion

An ALE meta-analysis of 275 studies provided a comprehensive overview of the brain regions involved in reasoning, revealing the extensive neural network engaged in this cognitive process. The reasoning process involves multiple brain regions, including the frontal, parietal, occipital, and temporal lobes, the limbic system, and subcortical areas, indicating that reasoning is a complex cognitive process requiring the coordination of several brain areas. The frontal regions, particularly the inferior, middle, and superior frontal gyri, are well known for their roles in higher cognitive functions, including planning, decision-making, and problem-solving (Fuster, 2001; Miller and Cohen, 2001). The parietal lobes, especially the inferior and superior parietal lobules, are crucial for the integration of sensory information and spatial reasoning (Culham et al., 2001). The occipital lobe and the fusiform gyrus are primarily involved in visual processing, which is essential for reasoning tasks involving visual stimuli or spatial manipulation (Grill-Spector and Malach, 2004; Kanwisher, 2010). The anterior cingulate cortex and the parahippocampal gyrus are involved in emotion regulation and memory, respectively (Bush et al., 2000; Eichenbaum, 2004), highlighting the importance of these functions in integrated reasoning (Dockès et al., 2020). Considering the brain regions and their corresponding functions, reasoning emerges as a complex higher-order function involving multiple cognitive processes.

The findings from 25 studies on the WCST further corroborate the broader findings of reasoning studies. The WCST is a widely used neuropsychological test that assesses executive functions, including cognitive flexibility, problem-solving, and abstract thinking (Grant and Berg, 1993). Although the WCST is primarily used to study executive functions, the peak activations in WCST studies align closely with those identified in the reasoning meta-analysis, suggesting that the cognitive processes involved in the WCST are closely related to reasoning abilities.

Gender-specific activation results indicate different neural networks for males and females in reasoning and WCST tasks, highlighting significant differences in brain activity patterns. In studies on female reasoning, significant activation areas are mainly concentrated in the deep brain regions of the limbic system, emphasizing regions associated with emotion processing, sensory information integration, and cognitive control (Bush et al., 2000; Eichenbaum, 2004). In contrast, studies on male reasoning show significant activation in the occipital, parietal, and parts of the frontal cortical areas, emphasizing regions involved in visuospatial processing, attention, and executive functions (Bush et al., 2000; Grill-Spector and Malach, 2004; Kanwisher, 2010). These differences suggest that males and females may employ different strategies or neural pathways to accomplish reasoning tasks. The WCST studies also reflect this gender difference to some extent.

Beyond describing the above gender results, we quantitatively compared gender differences in reasoning and tasks using GingerALE. The results showed that compared to males, females activate parts of the prefrontal and limbic systems, indicating that emotion processing is also involved in females’ handling of cognitive and complex decision-making tasks. This is consistent with reviews on gender in neuroscience (Cahill, 2006) and findings on gender in learning and memory (Andreano and Cahill, 2009). However, the “male minus female” results and gender differences driven by the WCST paradigm are corrected. Women may use more contextualized strategies in cognitive tasks, which affects brain region activation in executive function tasks (Miller and Halpern, 2014). In addition, the activity of the limbic system is related to differences in emotional processing and may affect women’s task performance (Laird et al., 2005a). Research has also found that women show stronger prefrontal cortex connectivity during certain tasks, which is associated with greater neural integration abilities (Prabhakaran et al., 1997). Finally, hormonal fluctuations have significant effects on women’s performance on emotional and executive function tasks (Bartley et al., 2018). This suggests that while there are significant differences in specific regions, the overall activation patterns between genders may not be as prominent when considering the entire neural network involved in these tasks. This partially supports studies indicating no significant gender differences in reasoning (Miller and Halpern, 2014).

## Data Availability

The original contributions presented in this study are included in this article/Supplementary material, further inquiries can be directed to the corresponding author.

## Footnotes

1
https://www.brainmap.org/sleuth/

2
http://www.brainmap.org/ale/

3
https://www.humanconnectome.org/software/connectome-workbench
