# Supplementary material for: Understanding gender differences in reasoning and specific paradigm using meta-analysis of neuroimaging
Source: Front Behav Neurosci. 2025 Jan 7;18:1457663. doi: 10.3389/fnbeh.2024.1457663 (PMC11747635; doi:10.3389/fnbeh.2024.1457663)
Supplement: Supplementary file 1 [file Table_1.DOC]

**Supplementary Material**

Supplementary Table 1. Significant clusters (FDR p < .05) of all reason studies revealed by the ALE analysis.

| Cluster | Volume (mm^3^) | ALE | Weighted Centre  x y z | Peaks | Peak MNI  x y z | | | Anatomical Region |
| --- | --- | --- | --- | --- | --- | --- | --- | --- |
| 1 | 119576 | 0.218 | -0.3 15.1 14.7 | 26 | -46 | 8 | 30 | Left Frontal Lobe, Inferior Frontal Gyrus, Brodmann area 9 |
|  |  |  |  |  | 34 | 22 | -4 | Right Sub-lobar |
|  |  |  |  |  | -32 | 22 | -2 | Left Sub-lobar |
|  |  |  |  |  | 48 | 12 | 28 | Right Frontal Lobe, Inferior Frontal Gyrus, Brodmann area 9 |
|  |  |  |  |  | -12 | 10 | -6 | Left Sub-lobar, Lentiform Nucleus |
|  |  |  |  |  | 48 | 34 | 26 | Right Frontal Lobe, Middle Frontal Gyrus, Brodmann area 9 |
|  |  |  |  |  | -44 | 30 | 28 | Left Frontal Lobe, Middle Frontal Gyrus, Brodmann area 9 |
|  |  |  |  |  | 12 | 10 | 0 | Right Sub-lobar, Caudate, Caudate Head |
|  |  |  |  |  | 28 | 4 | 52 | Right Frontal Lobe, Sub-Gyral, Brodmann area 6 |
|  |  |  |  |  | -26 | -2 | 54 | Left Frontal Lobe, Middle Frontal Gyrus, Brodmann area 6 |
|  |  |  |  |  | -14 | -4 | -4 | Left Sub-lobar, Lentiform Nucleus, Medial Globus Pallidus |
|  |  |  |  |  | -50 | 26 | -6 | Left Frontal Lobe, Inferior Frontal Gyrus, Brodmann area 47 |
|  |  |  |  |  | -44 | 32 | 14 | Left Frontal Lobe, Middle Frontal Gyrus, Brodmann area 46 |
|  |  |  |  |  | -22 | -4 | -16 | Left Limbic Lobe, Parahippocampal Gyrus, Amygdala |
|  |  |  |  |  | 32 | 14 | 54 | Right Frontal Lobe, Middle Frontal Gyrus, Brodmann area 6 |
|  |  |  |  |  | 10 | -10 | 8 | Right Sub-lobar, Thalamus |
|  |  |  |  |  | -36 | 52 | 10 | Left Frontal Lobe, Middle Frontal Gyrus, Brodmann area 10 |
|  |  |  |  |  | -10 | -18 | 10 | Left Sub-lobar, Thalamus, Medial Dorsal Nucleus |
|  |  |  |  |  | 52 | 26 | 0 | Right Frontal Lobe, Inferior Frontal Gyrus, Brodmann area 45 |
|  |  |  |  |  | 26 | -4 | -18 | Right Limbic Lobe, Parahippocampal Gyrus, Amygdala |
|  |  |  |  |  | 34 | 50 | 20 | Right Frontal Lobe, Middle Frontal Gyrus, Brodmann area 10 |
|  |  |  |  |  | 38 | 52 | -4 | Right Frontal Lobe, Middle Frontal Gyrus, Brodmann area 10 |
|  |  |  |  |  | -2 | -6 | 8 | Left Sub-lobar, Thalamus |
|  |  |  |  |  | 38 | 16 | 42 | Right Frontal Lobe, Middle Frontal Gyrus, Brodmann area 6 |
|  |  |  |  |  | -16 | 0 | 16 | Left Sub-lobar, Caudate, Caudate Body |
|  |  |  |  |  | 32 | -10 | 62 | Right Frontal Lobe, Precentral Gyrus, Brodmann area 6 |
| 2 | 44760 | 0.207 | -34.7 -62.2 31.1 | 15 | -28 | -62 | 48 | Left Parietal Lobe, Superior Parietal Lobule, Brodmann area 7 |
|  |  |  |  |  | -42 | -44 | 44 | Left Parietal Lobe, Inferior Parietal Lobule, Brodmann area 40 |
|  |  |  |  |  | -44 | -80 | -4 | Left Occipital Lobe, Inferior Occipital Gyrus, Brodmann area 19 |
|  |  |  |  |  | -32 | -86 | 4 | Left Occipital Lobe, Middle Occipital Gyrus, Brodmann area 18 |
|  |  |  |  |  | -40 | -60 | -16 | Left Cerebellum, Posterior Lobe, Declive |
|  |  |  |  |  | -46 | -62 | -10 | Left Temporal Lobe, Fusiform Gyrus, Brodmann area 37 |
|  |  |  |  |  | -48 | -56 | 30 | Left Temporal Lobe, Superior Temporal Gyrus, Brodmann area 39 |
|  |  |  |  |  | -28 | -84 | 28 | Left Occipital Lobe, Middle Occipital Gyrus, Brodmann area 19 |
|  |  |  |  |  | -28 | -90 | -6 | Left Occipital Lobe, Inferior Occipital Gyrus, Brodmann area 18 |
|  |  |  |  |  | -40 | -76 | 34 | Left Occipital Lobe, Superior Occipital Gyrus, Brodmann area 19 |
|  |  |  |  |  | -30 | -64 | -12 | Left Cerebellum, Posterior Lobe, Declive |
|  |  |  |  |  | -60 | -52 | 32 | Left Parietal Lobe, Supramarginal Gyrus, Brodmann area 40 |
|  |  |  |  |  | -26 | -84 | -10 | Left Occipital Lobe, Lingual Gyrus, Brodmann area 18 |
|  |  |  |  |  | -6 | -74 | 44 | Left Parietal Lobe, Precuneus, Brodmann area 7 |
|  |  |  |  |  | -58 | -32 | 40 | Left Parietal Lobe, Inferior Parietal Lobule, Brodmann area 40 |
| 3 | 36480 | 0.192 | 0.2 28 31.5 | 15 | 0 | 18 | 48 | Left Frontal Lobe, Superior Frontal Gyrus, Brodmann area 6 |
|  |  |  |  |  | 4 | 20 | 46 | Right Frontal Lobe, Medial Frontal Gyrus, Brodmann area 6 |
|  |  |  |  |  | -4 | 42 | -8 | Left Limbic Lobe, Anterior Cingulate, Brodmann area 24 |
|  |  |  |  |  | 2 | 48 | -10 | Right Limbic Lobe, Anterior Cingulate, Brodmann area 32 |
|  |  |  |  |  | 4 | 52 | 22 | Right Frontal Lobe, Medial Frontal Gyrus, Brodmann area 9 |
|  |  |  |  |  | -6 | 56 | 24 | Left Frontal Lobe, Superior Frontal Gyrus, Brodmann area 9 |
|  |  |  |  |  | -4 | -6 | 52 | Left Frontal Lobe, Medial Frontal Gyrus, Brodmann area 6 |
|  |  |  |  |  | -8 | 56 | 30 | Left Frontal Lobe, Medial Frontal Gyrus, Brodmann area 9 |
|  |  |  |  |  | 2 | 32 | 12 | Left Limbic Lobe, Anterior Cingulate, Brodmann area 24 |
|  |  |  |  |  | 8 | 34 | -8 | Right Limbic Lobe, Anterior Cingulate, Brodmann area 24 |
|  |  |  |  |  | -2 | 56 | 10 | Left Frontal Lobe, Medial Frontal Gyrus, Brodmann area 9 |
|  |  |  |  |  | -4 | 50 | 10 | Left Frontal Lobe, Medial Frontal Gyrus, Brodmann area 9 |
|  |  |  |  |  | 4 | -6 | 68 | Right Frontal Lobe, Medial Frontal Gyrus, Brodmann area 6 |
|  |  |  |  |  | 8 | 6 | 66 | Right Frontal Lobe, Superior Frontal Gyrus, Brodmann area 6 |
|  |  |  |  |  | -2 | 42 | 8 | Left Limbic Lobe, Anterior Cingulate, Brodmann area 32 |
| 4 | 24952 | 0.181 | 36.4 -54.6 45 | 6 | 32 | -58 | 50 | Right Parietal Lobe, Superior Parietal Lobule, Brodmann area 7 |
|  |  |  |  |  | 44 | -42 | 44 | Right Parietal Lobe, Inferior Parietal Lobule, Brodmann area 40 |
|  |  |  |  |  | 30 | -72 | 36 | Right Parietal Lobe, Precuneus, Brodmann area 19 |
|  |  |  |  |  | 18 | -66 | 54 | Right Parietal Lobe, Precuneus, Brodmann area 7 |
|  |  |  |  |  | -2 | -56 | 54 | Left Parietal Lobe, Precuneus, Brodmann area 7 |
|  |  |  |  |  | 10 | -70 | 44 | Right Parietal Lobe, Precuneus, Brodmann area 7 |
| 5 | 13640 | 0.110 | 33.4 -76.7 -4 | 12 | 36 | -84 | 16 | Right Occipital Lobe, Middle Occipital Gyrus, Brodmann area 19 |
|  |  |  |  |  | 46 | -56 | -16 | Right Temporal Lobe, Fusiform Gyrus, Brodmann area 37 |
|  |  |  |  |  | 26 | -88 | -8 | Right Occipital Lobe, Middle Occipital Gyrus, Brodmann area 18 |
|  |  |  |  |  | 42 | -70 | -10 | Right Occipital Lobe, Fusiform Gyrus, Brodmann area 19 |
|  |  |  |  |  | 40 | -64 | -10 | Right Cerebellum, Posterior Lobe, Declive |
|  |  |  |  |  | 32 | -72 | -8 | Right Occipital Lobe, Fusiform Gyrus, Brodmann area 19 |
|  |  |  |  |  | 26 | -94 | -2 | Right Occipital Lobe, Lingual Gyrus, Brodmann area 17 |
|  |  |  |  |  | 38 | -84 | 0 | Right Occipital Lobe, Inferior Occipital Gyrus, Brodmann area 18 |
|  |  |  |  |  | 18 | -80 | -12 | Right Cerebellum, Posterior Lobe, Declive |
|  |  |  |  |  | 4 | -86 | -2 | Right Occipital Lobe, Lingual Gyrus, Brodmann area 18 |
|  |  |  |  |  | 10 | -74 | -2 | Right Occipital Lobe, Lingual Gyrus, Brodmann area 18 |
|  |  |  |  |  | 10 | -84 | -8 | Right Occipital Lobe, Lingual Gyrus, Brodmann area 18 |
| 6 | 3360 | 0.104 | -3.7 -56.3 25.5 | 4 | -8 | -56 | 22 | Left Limbic Lobe, Posterior Cingulate, Brodmann area 23 |
|  |  |  |  |  | -2 | -58 | 34 | Left Limbic Lobe, Cingulate Gyrus, Brodmann area 31 |
|  |  |  |  |  | 8 | -52 | 34 | Right Parietal Lobe, Precuneus, Brodmann area 31 |
|  |  |  |  |  | 6 | -52 | 18 | Right Limbic Lobe, Posterior Cingulate, Brodmann area 30 |
| 7 | 2328 | 0.103 | 0.5 -27.3 33.1 | 1 | 0 | -28 | 34 | Left Limbic Lobe, Cingulate Gyrus, Brodmann area 31 |
| 8 | 1408 | 0.081 | -0.6 -23.6 -8.5 | 2 | -2 | -26 | -6 | Left Brainstem, Midbrain, Red Nucleus |
|  |  |  |  |  | 6 | -20 | -10 | Right Brainstem, Midbrain, Red Nucleus |
| 9 | 296 | 0.072 | -54.7 -39 -5.7 | 1 | -54 | -38 | -4 | Left Temporal Lobe, Middle Temporal Gyrus |
| 10 | 208 | 0.74 | -19.7 -17.8 67.2 | 1 | -18 | -16 | 68 | Left Frontal Lobe, Precentral Gyrus, Brodmann area 4 |

Supplementary Table 2. Significant clusters (FDR p < .05) of all WCST tasks revealed by the ALE analysis.

| Cluster | Volume (mm^3^) | ALE | Weighted Centre  x y z | Peaks | Peak MNI  x y z | | | Anatomical Region |
| --- | --- | --- | --- | --- | --- | --- | --- | --- |
| 1 | 11944 | 0.051 | -41.5 19.9 20.8 | 6 | -44 | 14 | 30 | Left Frontal Lobe, Inferior Frontal Gyrus, Brodmann area 9 |
|  |  |  |  |  | -32 | 22 | 0 | Left Sub-lobar, Claustrum |
|  |  |  |  |  | -44 | 22 | 24 | Left Frontal Lobe, Middle Frontal Gyrus, Brodmann area 46 |
|  |  |  |  |  | -48 | 28 | 26 | Left Frontal Lobe, Middle Frontal Gyrus, Brodmann area 9 |
|  |  |  |  |  | -46 | 24 | 32 | Left Frontal Lobe, Middle Frontal Gyrus, Brodmann area 9 |
|  |  |  |  |  | -42 | 36 | 14 | Left Frontal Lobe, Middle Frontal Gyrus, Brodmann area 46 |
| 2 | 8744 | 0.050 | -32.2 -58.1 45.2 | 5 | -28 | -62 | 50 | Left Parietal Lobe, Superior Parietal Lobule, Brodmann area 7 |
|  |  |  |  |  | -36 | -52 | 46 | Left Parietal Lobe, Inferior Parietal Lobule, Brodmann area 40 |
|  |  |  |  |  | -44 | -42 | 44 | Left Parietal Lobe, Inferior Parietal Lobule, Brodmann area 40 |
|  |  |  |  |  | -26 | -68 | 42 | Left Parietal Lobe, Precuneus, Brodmann area 7 |
|  |  |  |  |  | -26 | -80 | 28 | Left Occipital Lobe, Cuneus, Brodmann area 18 |
| 3 | 7184 | 0.054 | 33.8 -61.2 45.6 | 4 | 34 | -62 | 50 | Right Parietal Lobe, Superior Parietal Lobule, Brodmann area 7 |
|  |  |  |  |  | 34 | -52 | 42 | No Gray Matter found |
|  |  |  |  |  | 30 | -74 | 36 | Right Parietal Lobe, Precuneus, Brodmann area 19 |
|  |  |  |  |  | 38 | -72 | 38 | Right Parietal Lobe, Precuneus, Brodmann area 19 |
| 4 | 4416 | 0.054 | 2 22.3 46.4 | 1 | 2 | 22 | 48 | Left Frontal Lobe, Medial Frontal Gyrus, Brodmann area 6 |
| 5 | 4008 | 0.035 | 42.8 12.6 36.1 | 4 | 46 | 14 | 28 | Right Frontal Lobe, Precentral Gyrus, Brodmann area 9 |
|  |  |  |  |  | 38 | 16 | 42 | Right Frontal Lobe, Middle Frontal Gyrus, Brodmann area 6 |
|  |  |  |  |  | 36 | 12 | 56 | Right Frontal Lobe, Middle Frontal Gyrus, Brodmann area 6 |
|  |  |  |  |  | 48 | 4 | 20 | Right Frontal Lobe, Inferior Frontal Gyrus, Brodmann area 9 |
| 6 | 3136 | 0.051 | 33 22.9 -3.2 | 1 | 32 | 24 | -4 | Right Sub-lobar, Claustrum |
| 7 | 2888 | 0.032 | -32.7 -81.9 -4.7 | 5 | -26 | -88 | 0 | Left Occipital Lobe, Middle Occipital Gyrus, Brodmann area 18 |
|  |  |  |  |  | -40 | -86 | 0 | Left Occipital Lobe, Inferior Occipital Gyrus, Brodmann area 19 |
|  |  |  |  |  | -36 | -70 | -12 | Left Cerebellum, Posterior Lobe, Declive |
|  |  |  |  |  | -40 | -74 | -12 | Left Occipital Lobe, Fusiform Gyrus, Brodmann area 19 |
|  |  |  |  |  | -34 | -88 | 10 | Left Occipital Lobe, Middle Occipital Gyrus, Brodmann area 19 |
| 8 | 2400 | 0.033 | 12.6 -0.9 1.5 | 3 | 10 | -12 | 6 | Right Sub-lobar, Thalamus, Medial Dorsal Nucleus |
|  |  |  |  |  | 14 | 12 | 0 | Right Sub-lobar, Caudate, Caudate Head |
|  |  |  |  |  | 14 | 0 | -2 | Right Sub-lobar, Lentiform Nucleus, Medial Globus Pallidus |
| 9 | 2264 | 0.037 | -12.3 0.1 4.3 | 4 | -10 | -6 | 4 | Left Sub-lobar, Thalamus, Ventral Lateral Nucleus |
|  |  |  |  |  | -18 | 6 | 0 | Left Sub-lobar, Lentiform Nucleus, Putamen |
|  |  |  |  |  | -10 | 8 | 4 | Left Sub-lobar, Caudate, Caudate Body |
|  |  |  |  |  | -14 | 10 | 8 | Left Sub-lobar, Caudate, Caudate Body |
| 10 | 2160 | 0.043 | 34.6 -88.5 -4.9 | 3 | 34 | -88 | -10 | Right Occipital Lobe, Fusiform Gyrus, Brodmann area 19 |
|  |  |  |  |  | 34 | -90 | 2 | Right Occipital Lobe, Middle Occipital Gyrus, Brodmann area 18 |
|  |  |  |  |  | 28 | -94 | -14 | Right Occipital Lobe, Fusiform Gyrus, Brodmann area 18 |
| 11 | 2160 | 0.035 | 43.6 32.3 27.2 | 4 | 42 | 30 | 28 | Right Frontal Lobe, Middle Frontal Gyrus, Brodmann area 9 |
|  |  |  |  |  | 40 | 40 | 18 | Right Frontal Lobe, Middle Frontal Gyrus, Brodmann area 9 |
|  |  |  |  |  | 44 | 40 | 24 | Right Frontal Lobe, Superior Frontal Gyrus, Brodmann area 9 |
|  |  |  |  |  | 44 | 34 | 16 | Right Frontal Lobe, Middle Frontal Gyrus, Brodmann area 46 |
| 12 | 1328 | 0.033 | -31.1 56 10.2 | 1 | -32 | 56 | 12 | Left Frontal Lobe, Middle Frontal Gyrus, Brodmann area 10 |
| 13 | 496 | 0.027 | 9.7 -80.6 -20 | 2 | 10 | -80 | -18 | Right Cerebellum, Posterior Lobe, Declive |
|  |  |  |  |  | 10 | -82 | -28 | Right Cerebellum, Posterior Lobe, Pyramis |
| 14 | 344 | 0.021 | -34.2 53.8 -2.5 | 1 | -34 | 56 | -2 | Left Frontal Lobe, Middle Frontal Gyrus, Brodmann area 10 |
| 15 | 296 | 0.023 | 47.2 -46.3 38.7 | 1 | 48 | -46 | 38 | Right Parietal Lobe, Supramarginal Gyrus, Brodmann area 40 |
| 16 | 248 | 0.022 | 32.8 55 3 | 2 | 32 | 54 | 4 | Right Frontal Lobe, Middle Frontal Gyrus, Brodmann area 10 |
|  |  |  |  |  | 32 | 54 | -4 | Right Frontal Lobe, Middle Frontal Gyrus, Brodmann area 10 |
| 17 | 224 | 0.024 | 39 15 -28.7 | 1 | 38 | 16 | -30 | Right Temporal Lobe, Superior Temporal Gyrus, Brodmann area 38 |
| 18 | 200 | 0.024 | -21.1 -43.6 54 | 1 | -22 | -44 | 54 | Left Parietal Lobe, Precuneus, Brodmann area 7 |

Supplementary Table 3. Significant clusters (FDR p < .05) of all reason studies about females revealed by the ALE analysis.

| Cluster | Volume (mm^3^) | ALE | Weighted Centre  x y z | Peaks | Peak MNI  x y z | | | Anatomical Region |
| --- | --- | --- | --- | --- | --- | --- | --- | --- |
| 1 | 1664 | 0.035 | 34.5 24.4 3.5 | 1 | 34 | 24 | 6 | Right Sub-lobar, Insula, Brodmann area 13 |
| 2 | 1552 | 0.033 | -42.9 -43.1 45 | 1 | -42 | -42 | 46 | Left Parietal Lobe, Inferior Parietal Lobule, Brodmann area 40 |
| 3 | 1504 | 0.029 | 5 25.5 34.1 | 3 | 8 | 24 | 32 | Right Limbic Lobe, Cingulate Gyrus, Brodmann area 32 |
|  |  |  |  |  | 4 | 26 | 38 | Right Limbic Lobe, Cingulate Gyrus, Brodmann area 32 |
|  |  |  |  |  | -6 | 24 | 32 | Left Limbic Lobe, Cingulate Gyrus, Brodmann area 32 |
| 4 | 1376 | 0.036 | 46.8 -61.9 -4.3 | 3 | 46 | -58 | -2 | Right Temporal Lobe, Middle Temporal Gyrus, Brodmann area 37 |
|  |  |  |  |  | 44 | -70 | -12 | Right Occipital Lobe, Fusiform Gyrus, Brodmann area 19 |
|  |  |  |  |  | 50 | -66 | -10 | Right Temporal Lobe, Fusiform Gyrus, Brodmann area 37 |
| 5 | 832 | 0.026 | 9.2 13.3 47.3 | 1 | 10 | 12 | 50 | Right Frontal Lobe, Medial Frontal Gyrus, Brodmann area 6 |
| 6 | 712 | 0.028 | 48.2 10.6 24.6 | 1 | 48 | 10 | 26 | Right Frontal Lobe, Inferior Frontal Gyrus, Brodmann area 9 |
| 7 | 688 | 0.025 | -47.1 -60.3 -10.9 | 2 | -48 | -58 | -14 | Left Temporal Lobe, Fusiform Gyrus, Brodmann area 37 |
|  |  |  |  |  | -46 | -66 | -6 | Left Temporal Lobe, Fusiform Gyrus, Brodmann area 37 |
| 8 | 688 | 0.027 | 5.6 -49.6 36.7 | 1 | 8 | -50 | 36 | Right Parietal Lobe, Precuneus, Brodmann area 31 |
| 9 | 632 | 0.027 | -46.7 39 20.9 | 1 | -48 | 40 | 22 | Left Frontal Lobe, Middle Frontal Gyrus, Brodmann area 46 |
| 10 | 624 | 03029 | -20.1 6.7 4 | 1 | -20 | 6 | 4 | Left Sub-lobar, Lentiform Nucleus, Putamen |
| 11 | 584 | 0.021 | -48.5 -0.3 33.8 | 2 | -52 | 0 | 32 | Left Frontal Lobe, Precentral Gyrus, Brodmann area 6 |
|  |  |  |  |  | -44 | -4 | 36 | Left Frontal Lobe, Precentral Gyrus, Brodmann area 6 |
| 12 | 528 | 0.024 | -46.4 -3.7 -16.2 | 2 | -44 | -4 | -20 | Left Temporal Lobe, Sub-Gyral, Brodmann area 21 |
|  |  |  |  |  | -50 | -4 | -10 | Left Temporal Lobe, Superior Temporal Gyrus, Brodmann area 22 |
| 13 | 480 | 0.023 | 43.6 -46 45.7 | 2 | 42 | -46 | 42 | Right Parietal Lobe, Inferior Parietal Lobule, Brodmann area 40 |
|  |  |  |  |  | 46 | -46 | 50 | Right Parietal Lobe, Inferior Parietal Lobule, Brodmann area 40 |
| 14 | 456 | 0.023 | 28.9 -6.8 53.2 | 2 | 30 | -4 | 54 | Right Frontal Lobe, Middle Frontal Gyrus, Brodmann area 6 |
|  |  |  |  |  | 28 | -10 | 52 | Right Frontal Lobe, Precentral Gyrus, Brodmann area 6 |
| 15 | 432 | 0.025 | 16.2 7.6 5.8 | 1 | 16 | 8 | 6 | Right Sub-lobar, Caudate, Caudate Body |
| 16 | 424 | 0.025 | 4.3 -13.9 -11.3 | 1 | 2 | -14 | -12 | Left Brainstem, Midbrain, Mammillary Body |
| 17 | 400 | 0.025 | -21.9 -56.9 43.2 | 1 | -22 | -58 | 44 | Left Parietal Lobe, Precuneus, Brodmann area 7 |
| 18 | 360 | 0.024 | 11.5 48.6 8.9 | 1 | 10 | 50 | 8 | Right Frontal Lobe, Medial Frontal Gyrus, Brodmann area 9 |
| 19 | 360 | 0.022 | 47.3 38.3 25.1 | 1 | 48 | 42 | 28 | Right Frontal Lobe, Superior Frontal Gyrus, Brodmann area 9 |
| 20 | 344 | 0.025 | -10 48.8 3.4 | 1 | -10 | 48 | 4 | Left Frontal Lobe, Medial Frontal Gyrus, Brodmann area 10 |
| 21 | 312 | 0.026 | 42.3 -4.1 -14.6 | 1 | 42 | -4 | -14 | Right Sub-lobar, Claustrum |
| 22 | 272 | 0.022 | -34.6 20.7 1.5 | 1 | -34 | 20 | 2 | Left Sub-lobar, Insula, Brodmann area 13 |
| 23 | 272 | 0.022 | -23.5 -79.3 37.2 | 1 | -24 | -80 | 36 | Left Occipital Lobe, Precuneus, Brodmann area 31 |
| 24 | 264 | 0.023 | -5.4 6.5 46.3 | 1 | -6 | 6 | 46 | Left Limbic Lobe, Cingulate Gyrus, Brodmann area 24 |
| 25 | 224 | 0.024 | -4.6 39.7 -2 | 1 | -4 | 40 | -2 | Left Limbic Lobe, Anterior Cingulate, Brodmann area 24 |
| 26 | 224 | 0.022 | 8.9 -75.1 19.1 | 1 | 10 | -76 | 18 | Right Occipital Lobe, Cuneus, Brodmann area 23 |
| 27 | 200 | 0.022 | -43.3 15.7 33.8 | 1 | -44 | 16 | 34 | Left Frontal Lobe, Middle Frontal Gyrus, Brodmann area 9 |

Supplementary Table 4. Significant clusters (FDR p < .05) of all reason studies about males revealed by the ALE analysis.

| Cluster | Volume (mm^3^) | ALE | Weighted Centre  x y z | Peaks | Peak MNI  x y z | | | Anatomical Region |
| --- | --- | --- | --- | --- | --- | --- | --- | --- |
| 1 | 12464 | 0.078 | -32.8 -55.1 46 | 6 | -38 | -44 | 46 | *Left Parietal Lobe, Inferior Parietal Lobule, Brodmann area 40* |
|  |  |  |  |  | -32 | -54 | 50 | Left Parietal Lobe, Inferior Parietal Lobule, Brodmann area 7 |
|  |  |  |  |  | -22 | -70 | 52 | Left Parietal Lobe, Superior Parietal Lobule, Brodmann area 7 |
|  |  |  |  |  | -24 | -78 | 40 | Left Parietal Lobe, Precuneus, Brodmann area 19 |
|  |  |  |  |  | -10 | -72 | 50 | Left Parietal Lobe, Precuneus, Brodmann area 7 |
|  |  |  |  |  | -52 | -34 | 46 | Left Parietal Lobe, Inferior Parietal Lobule, Brodmann area 40 |
| 2 | 11128 | 0.064 | 37.2 -54.5 45.8 | 7 | 46 | -42 | 48 | Right Parietal Lobe, Inferior Parietal Lobule, Brodmann area 40 |
|  |  |  |  |  | 30 | -68 | 40 | Right Parietal Lobe, Precuneus, Brodmann area 7 |
|  |  |  |  |  | 32 | -50 | 42 | No Gray Matter found |
|  |  |  |  |  | 34 | -62 | 50 | Right Parietal Lobe, Superior Parietal Lobule, Brodmann area 7 |
|  |  |  |  |  | 30 | -68 | 30 | Right Occipital Lobe, Precuneus, Brodmann area 31 |
|  |  |  |  |  | 46 | -56 | 48 | Right Parietal Lobe, Inferior Parietal Lobule, Brodmann area 40 |
|  |  |  |  |  | 22 | -72 | 52 | Right Parietal Lobe, Precuneus, Brodmann area 7 |
| 3 | 8576 | 0.097 | 36.8 24.4 -0.2 | 7 | 34 | 26 | -4 | Right Sub-lobar, Insula, Brodmann area 13 |
|  |  |  |  |  | 34 | 24 | 6 | Right Sub-lobar, Insula, Brodmann area 13 |
|  |  |  |  |  | 48 | 16 | 4 | Right Frontal Lobe, Precentral Gyrus, Brodmann area 44 |
|  |  |  |  |  | 34 | 38 | 8 | Right Frontal Lobe, Middle Frontal Gyrus, Brodmann area 10 |
|  |  |  |  |  | 46 | 40 | 2 | Right Frontal Lobe, Inferior Frontal Gyrus, Brodmann area 46 |
|  |  |  |  |  | 34 | 42 | 6 | Right Frontal Lobe, Middle Frontal Gyrus, Brodmann area 10 |
|  |  |  |  |  | 52 | 34 | 0 | Right Frontal Lobe, Inferior Frontal Gyrus, Brodmann area 46 |
| 4 | 7816 | 0.067 | 0.5 19 44.4 | 6 | 0 | 28 | 40 | Left Limbic Lobe, Cingulate Gyrus, Brodmann area 32 |
|  |  |  |  |  | 8 | 14 | 50 | Right Frontal Lobe, Superior Frontal Gyrus, Brodmann area 6 |
|  |  |  |  |  | -4 | 22 | 46 | Left Frontal Lobe, Medial Frontal Gyrus, Brodmann area 6 |
|  |  |  |  |  | -4 | 6 | 48 | Left Limbic Lobe, Cingulate Gyrus, Brodmann area 24 |
|  |  |  |  |  | -2 | 12 | 58 | Left Frontal Lobe, Superior Frontal Gyrus, Brodmann area 6 |
|  |  |  |  |  | 10 | 34 | 32 | Right Frontal Lobe, Medial Frontal Gyrus, Brodmann area 6 |
| 5 | 4048 | 0.054 | -36.3 22.4 -2.4 | 2 | -32 | 22 | 2 | Left Sub-lobar, Insula, Brodmann area 13 |
|  |  |  |  |  | -46 | 22 | -4 | Left Frontal Lobe, Inferior Frontal Gyrus, Brodmann area 47 |
| 6 | 3736 | 0.052 | -13 8 2.2 | 5 | -10 | 8 | -6 | Left Sub-lobar, Caudate, Caudate Head |
|  |  |  |  |  | -18 | 4 | 14 | Left Sub-lobar, Caudate, Caudate Body |
|  |  |  |  |  | -10 | 14 | 4 | Left Sub-lobar, Caudate, Caudate Body |
|  |  |  |  |  | -20 | 6 | 2 | Left Sub-lobar, Lentiform Nucleus, Putamen |
|  |  |  |  |  | -4 | 12 | 4 | Left Sub-lobar, Caudate, Caudate Body |
| 7 | 3608 | 0.055 | 14.6 8.7 2.2 | 3 | 16 | 6 | 2 | Right Sub-lobar, Lentiform Nucleus, Lateral Globus Pallidus |
|  |  |  |  |  | 12 | 8 | 0 | Right Sub-lobar, Caudate, Caudate Head |
|  |  |  |  |  | 18 | 16 | 6 | Right Sub-lobar, Caudate, Caudate Body |
| 8 | 3536 | 0.046 | -46 4.8 31.1 | 1 | -44 | 6 | 30 | Left Frontal Lobe, Inferior Frontal Gyrus, Brodmann area 9 |
| 9 | 3472 | 0.046 | -43.6 29.7 25.3 | 3 | -44 | 30 | 30 | Left Frontal Lobe, Middle Frontal Gyrus, Brodmann area 9 |
|  |  |  |  |  | -42 | 34 | 24 | Left Frontal Lobe, Middle Frontal Gyrus, Brodmann area 9 |
|  |  |  |  |  | -44 | 22 | 16 | Left Frontal Lobe, Middle Frontal Gyrus, Brodmann area 46 |
| 10 | 3336 | 0.057 | 50.2 11.2 26.9 | 1 | 50 | 12 | 26 | Right Frontal Lobe, Inferior Frontal Gyrus, Brodmann area 9 |
| 11 | 2896 | 0.055 | 47.9 -64.3 -6.9 | 3 | 50 | -62 | -8 | Right Temporal Lobe, Fusiform Gyrus, Brodmann area 37 |
|  |  |  |  |  | 46 | -74 | -6 | Right Occipital Lobe, Inferior Occipital Gyrus, Brodmann area 19 |
|  |  |  |  |  | 42 | -70 | -14 | Right Occipital Lobe, Fusiform Gyrus, Brodmann area 19 |
| 12 | 2728 | 0.060 | 44.2 36.5 25.8 | 2 | 46 | 36 | 28 | Right Frontal Lobe, Middle Frontal Gyrus, Brodmann area 9 |
|  |  |  |  |  | 48 | 40 | 14 | Right Frontal Lobe, Middle Frontal Gyrus, Brodmann area 46 |
| 13 | 2008 | 0.040 | -5.9 -15.5 2.5 | 3 | -8 | -22 | 14 | Left Sub-lobar, Thalamus |
|  |  |  |  |  | -4 | -14 | -6 | Left Sub-lobar, Thalamus |
|  |  |  |  |  | -8 | -12 | 6 | Left Sub-lobar, Thalamus, Medial Dorsal Nucleus |
| 14 | 1568 | 0.055 | 29 -89.6 -5.3 | 2 | 30 | -90 | -6 | Right Occipital Lobe, Middle Occipital Gyrus, Brodmann area 18 |
|  |  |  |  |  | 18 | -84 | -8 | Right Occipital Lobe, Lingual Gyrus, Brodmann area 18 |
| 15 | 1400 | 0.039 | -26.9 0.1 53.7 | 2 | -26 | -2 | 54 | Left Frontal Lobe, Middle Frontal Gyrus, Brodmann area 6 |
|  |  |  |  |  | -30 | 8 | 52 | Left Frontal Lobe, Middle Frontal Gyrus, Brodmann area 6 |
| 16 | 1360 | 0.042 | 30.8 -2.2 59.5 | 1 | 30 | -4 | 60 | Right Frontal Lobe, Middle Frontal Gyrus, Brodmann area 6 |
| 17 | 1248 | 0.042 | -39.9 -71.7 -12.8 | 2 | -42 | -72 | -16 | Left Cerebellum, Posterior Lobe, Declive |
|  |  |  |  |  | -46 | -68 | -4 | Left Occipital Lobe, Middle Occipital Gyrus, Brodmann area 37 |
| 18 | 752 | 0.038 | -28.5 -83.8 -6.7 | 1 | -28 | -84 | -6 | Left Occipital Lobe, Middle Occipital Gyrus, Brodmann area 18 |
| 19 | 680 | 0.037 | -9.1 -92.1 -3.7 | 1 | -12 | -92 | -4 | Left Occipital Lobe, Inferior Occipital Gyrus, Brodmann area 17 |
| 20 | 584 | 0.043 | -49 -60.4 -13.2 | 1 | -50 | -60 | -14 | Left Temporal Lobe, Fusiform Gyrus, Brodmann area 37 |
| 21 | 576 | 0.041 | -10.9 -62.5 57.3 | 2 | -8 | -62 | 56 | Left Parietal Lobe, Precuneus, Brodmann area 7 |
|  |  |  |  |  | -14 | -62 | 60 | Left Parietal Lobe, Superior Parietal Lobule, Brodmann area 7 |
| 22 | 480 | 0.038 | 12.8 49.3 6 | 1 | 12 | 50 | 6 | Right Frontal Lobe, Medial Frontal Gyrus, Brodmann area 10 |
| 23 | 448 | 0.040 | 38.6 55.5 1.2 | 1 | 38 | 56 | 2 | Right Frontal Lobe, Middle Frontal Gyrus, Brodmann area 10 |
| 24 | 400 | 0.035 | -13.5 -78.4 7.9 | 1 | -16 | -78 | 6 | Left Occipital Lobe, Lingual Gyrus |
| 25 | 384 | 0.032 | -24.9 -85.1 18.8 | 2 | -28 | -82 | 20 | Left Occipital Lobe, Middle Occipital Gyrus, Brodmann area 19 |
|  |  |  |  |  | -22 | -88 | 18 | Left Occipital Lobe, Middle Occipital Gyrus, Brodmann area 18 |
| 26 | 312 | 0.040 | 25.5 0.3 -16.1 | 1 | 26 | 0 | -16 | Right Limbic Lobe, Parahippocampal Gyrus, Brodmann area 34 |
| 27 | 288 | 0.037 | -30.8 -65 -29.9 | 1 | -30 | -66 | -30 | Left Cerebellum, Posterior Lobe, Pyramis |
| 28 | 232 | 0.035 | 31.2 -47.6 -13.8 | 1 | 30 | -48 | -14 | Right Cerebellum, Anterior Lobe, Culmen |
| 29 | 208 | 0.034 | 39.8 -58.8 -15.8 | 1 | 40 | -58 | -16 | Right Cerebellum, Posterior Lobe, Declive |
| 30 | 208 | 0.034 | -49.8 -60.5 28.2 | 1 | -50 | -62 | 28 | Left Temporal Lobe, Middle Temporal Gyrus, Brodmann area 39 |

Supplementary Table 5. Significant clusters (FDR p < .05) of WCST tasks about females revealed by the ALE analysis.

| Cluster | Volume (mm^3^) | ALE | Weighted Centre  x y z | Peaks | Peak MNI  x y z | | | Anatomical Region |
| --- | --- | --- | --- | --- | --- | --- | --- | --- |
| 1 | 1232 | 0.020 | -5.3 23.3 36.3 | 3 | -8 | 22 | 34 | Left Limbic Lobe, Cingulate Gyrus, Brodmann area 32 |
|  |  |  |  |  | 4 | 26 | 40 | Right Limbic Lobe, Cingulate Gyrus, Brodmann area 32 |
|  |  |  |  |  | -6 | 18 | 44 | Left Frontal Lobe, Medial Frontal Gyrus, Brodmann area 32 |
| 2 | 1136 | 0.019 | 36.4 18.7 1 | 1 | 38 | 18 | 0 | Right Sub-lobar, Claustrum |
| 3 | 872 | 0.021 | -16.1 7 0.9 | 1 | -16 | 8 | 0 | Left Sub-lobar, Lentiform Nucleus, Putamen |
| 4 | 672 | 0.020 | 48 38 -13.8 | 1 | 48 | 38 | -14 | Right Frontal Lobe, Middle Frontal Gyrus, Brodmann area 47 |
| 5 | 440 | 0.018 | -5.4 -61.3 54.1 | 1 | -6 | -62 | 54 | Left Parietal Lobe, Precuneus, Brodmann area 7 |
| 6 | 360 | 0.018 | -29.5 -47.5 29.2 | 1 | -30 | -48 | 30 | No Gray Matter found |
| 7 | 224 | 0.015 | 27 -50 0 | 1 | 27 | -50 | 0 | Right Limbic Lobe, Parahippocampal Gyrus, Brodmann area 19 |
| 8 | 208 | 0.015 | -9.2 -40.1 0.1 | 1 | -10 | -40 | 0 | Left Limbic Lobe, Parahippocampal Gyrus, Brodmann area 30 |

Supplementary Table 6. Significant clusters (FDR p < .05) of WCST tasks about males revealed by the ALE analysis.

| Cluster | Volume (mm^3^) | ALE | Weighted Centre  x y z | Peaks | Peak MNI  x y z | | | Anatomical Region |
| --- | --- | --- | --- | --- | --- | --- | --- | --- |
| 1 | 1672 | 0.020 | -32.8 -56.6 47.6 | 3 | -26 | -60 | 48 | Left Parietal Lobe, Superior Parietal Lobule, Brodmann area 7 |
|  |  |  |  |  | -40 | -52 | 46 | Left Parietal Lobe, Inferior Parietal Lobule, Brodmann area 40 |
|  |  |  |  |  | -34 | -56 | 50 | Left Parietal Lobe, Inferior Parietal Lobule, Brodmann area 7 |
| 2 | 1040 | 0.018 | -18.2 -74 -24.3 | 2 | -16 | -72 | -24 | Left Cerebellum, Posterior Lobe, Uvula |
|  |  |  |  |  | -20 | -76 | -26 | Left Cerebellum, Posterior Lobe, Uvula |
| 3 | 984 | 0.019 | 39.8 -60.4 46.3 | 2 | 40 | -62 | 46 | Right Parietal Lobe, Inferior Parietal Lobule, Brodmann area 39 |
|  |  |  |  |  | 36 | -62 | 48 | Right Parietal Lobe, Precuneus, Brodmann area 19 |
| 4 | 720 | 0.017 | 26.5 -71.9 30.3 | 4 | 28 | -68 | 28 | Right Occipital Lobe, Precuneus, Brodmann area 31 |
|  |  |  |  |  | 24 | -80 | 30 | Right Occipital Lobe, Cuneus, Brodmann area 18 |
|  |  |  |  |  | 28 | -72 | 36 | Right Parietal Lobe, Precuneus, Brodmann area 19 |
|  |  |  |  |  | 26 | -74 | 32 | Right Occipital Lobe, Precuneus, Brodmann area 31 |
| 5 | 696 | 0.017 | -43.7 20.9 19 | 1 | -44 | 20 | 18 | Left Frontal Lobe, Middle Frontal Gyrus, Brodmann area 46 |
| 6 | 528 | 0.017 | -32.9 -68.8 -11.4 | 1 | -34 | -70 | -12 | Left Cerebellum, Posterior Lobe, Declive |
| 7 | 528 | 0.016 | -50.3 17.3 28.5 | 1 | -50 | 16 | 28 | Left Frontal Lobe, Inferior Frontal Gyrus, Brodmann area 9 |
| 8 | 504 | 0.016 | -25.2 -77.5 42.7 | 3 | -24 | -78 | 44 | Left Parietal Lobe, Precuneus, Brodmann area 19 |
|  |  |  |  |  | -26 | -78 | 40 | Left Parietal Lobe, Precuneus, Brodmann area 19 |
|  |  |  |  |  | -28 | -72 | 46 | Left Parietal Lobe, Precuneus, Brodmann area 19 |
| 9 | 488 | 0.019 | -35.7 55.6 12.9 | 1 | -36 | 56 | 12 | Left Frontal Lobe, Superior Frontal Gyrus, Brodmann area 10 |
| 10 | 464 | 0.014 | -33.1 -51.1 36.8 | 2 | -36 | -50 | 36 | Left Parietal Lobe, Supramarginal Gyrus, Brodmann area 40 |
|  |  |  |  |  | -28 | -54 | 36 | Left Temporal Lobe, Middle Temporal Gyrus, Brodmann area 39 |
| 11 | 448 | 0.016 | -28.4 -83 -4.3 | 1 | -28 | -82 | -6 | Left Occipital Lobe, Lingual Gyrus, Brodmann area 18 |
| 12 | 448 | 0.018 | 33.7 -50.6 40.5 | 1 | 34 | -50 | 40 | No Gray Matter found |
| 13 | 400 | 0.017 | -6 -86.1 35.5 | 1 | -6 | -86 | 36 | Left Occipital Lobe, Cuneus, Brodmann area 19 |
| 14 | 376 | 0.016 | -7.2 -77.2 43.4 | 1 | -8 | -76 | 44 | Left Parietal Lobe, Precuneus, Brodmann area 7 |
| 15 | 352 | 0.016 | -3 -97.4 -2.4 | 1 | -4 | -98 | -4 | Left Occipital Lobe, Lingual Gyrus, Brodmann area 17 |
| 16 | 352 | 0.017 | -26.7 -87.5 9 | 1 | -26 | -88 | 10 | Left Occipital Lobe, Middle Occipital Gyrus, Brodmann area 18 |
| 17 | 344 | 0.016 | -41.1 32.7 24.4 | 1 | -40 | 34 | 24 | Left Frontal Lobe, Middle Frontal Gyrus, Brodmann area 9 |
| 18 | 312 | 0.016 | -9 -70.6 -2 | 1 | -8 | -70 | -2 | Left Cerebellum, Anterior Lobe, Culmen |
| 19 | 296 | 0.016 | -1.2 3.4 53.7 | 1 | -2 | 4 | 54 | Left Frontal Lobe, Medial Frontal Gyrus, Brodmann area 6 |
| 20 | 216 | 0.015 | 21.8 -58.5 6.4 | 1 | 22 | -58 | 6 | Right Occipital Lobe, Lingual Gyrus, Brodmann area 18 |
| 21 | 208 | 0.014 | 52.1 12.4 30.3 | 1 | 52 | 12 | 30 | Right Frontal Lobe, Inferior Frontal Gyrus, Brodmann area 9 |
